# Youth Wellness in Relation to Attention-Deficit Hyperactivity Disorder and Behavior Problems

**DOI:** 10.1192/j.eurpsy.2026.10195

**Published:** 2026-07-31

**Authors:** K. Lin, S. A. Plenz, M. Grados

**Affiliations:** 1 Johns Hopkins University; 2 Johns Hopkins School of Medicine, Baltimore, United States

## Abstract

**Introduction:**

*These authors contributed equally. Parameters of wellness in youth include physical activity, sleep, and screen time. While these appear to be dysregulated in disruptive disorders such as attention-deficit hyperactivity disorder (ADHD) and behavior problems, the effect of ADHD on behavior problems and wellness parameters is not clearly understood.

**Objectives:**

To examine the effect of ADHD on wellness parameters in youth with behavior problems measured on a gradient (low, moderate, severe).

**Methods:**

The National Survey of Children’s Health (NSCH), a nationally representative sample of youth in the US for years 2022-2023, constituted the sample frame. Inclusion criteria were: 1) ages 5-17 years; 2) positive behavior problems (mild, moderate, severe); 3) ADHD data present (no/yes). Exclusion criteria were: 1) autism; 2) intellectual disability/developmental delay; 3) organic brain disease (epilepsy, cerebral palsy, Tourette syndrome). Six predictor groups were formed based on the severity of the behavior problems (3 levels) by presence of ADHD (yes/no). Multinomial logistic regressions were used to compare the predictive value of each of the five remaining groups using No ADHD, Mild Behavior Problems as a baseline group. The normative (most common) sleep, screen time and exercise values were also used as the baseline subgroup within the baseline group (No ADHD, Mild Behavior Problems). Chi-square tests were used for frequency data, and logistic regressions adjusted for socioeconomic status, caregiver mental health, and adversity measures.

**Results:**

From 109,265 households, only 2,690 children met inclusion criteria (10.77 +/- 3.31 years; 67% male). The severe behavior problems group was significantly less white (p < 0.001) and lower income (p < 0.001). Unexpectedly, the group with Severe Behavior Problems and No ADHD was the most discrepant from baseline, showing the worst wellness parameters. This group was ~3× more likely to report no physical activity (RRR=3.25 [1.34-7.88]) and 5× more likely to sleep less than 6 hours (RRR=5.00 [1.14-21.98]. Interestingly, screentime use depended on having ADHD compared to baseline (RRR= 1.80 [1.06–3.03] p < 0.05). Adjusting for confounders reduced, but did not eliminate, these effects.

**Conclusions:**

Behavior problems, especially in the severe range, have the greatest effect on wellness parameters in youth. ADHD modifies, but does not determine, worse wellness parameters. This finding highlights the immense impact of behavioral problems beyond ADHD, suggesting that targeted approaches to address family dysfunction, implement behavioral management and engage youth in productive activities early in life will diminish poor wellness beyond the effect of ADHD.

**Disclosure of Interest:**

None Declared

